# Deep impact? Is mercury in dab (*Limanda limanda*) a marker for dumped munition? Results from munition dump site Kolberger Heide (Baltic Sea)

**DOI:** 10.1007/s10661-021-09564-3

**Published:** 2021-11-10

**Authors:** Ulrike Kammann, Marc-Oliver Aust, Maike Siegmund, Nicole Schmidt, Katharina Straumer, Thomas Lang

**Affiliations:** Thünen Institute of Fisheries Ecology, Herwigstraße 31, Bremerhaven, 27572 Germany

**Keywords:** Bioaccumulation, Pollution, Fish, Age

## Abstract

Dumped munitions contain various harmful substances which can affect marine biota like fish. One of them is mercury (Hg), included in the common explosive primer Hg fulminate. There is still a lack of knowledge whether dumped munitions impact the Hg concentrations in the Baltic Sea environment. This study aims to answer the question if dab caught at the dump site Kolberger Heide show higher Hg concentrations released from munition sources and whether Hg in fish is a usable marker for munition exposure. Therefore, a total of 251 individual dab (Limanda limanda) were analysed including 99 fish from the dump site. In fish from the Kolberger Heide, no elevated Hg concentrations were found compared to reference sites when age-dependent bioaccumulation of mercury was considered. Therefore we conclude that Hg in fish is no suitable indicator for exposure to munition dumping, e.g. in the frame of possible future monitoring studies as Hg exposure originating from dumped munition is only a small contributor to overall Hg exposure of fish.

## Introduction

Dumped munition in the sea is a global problem and its management is a challenge for the future. After World War II, about 300,000 t of conventional munitions have been dumped in German coastal waters of the Baltic Sea inside eight official munition dump sites, often close to the coast (Beck et al., 2018). One of these sites is Kolberger Heide (KH), on the one hand an area in the Kiel Bay in about 2 km distance to the beach and the route of shipping traffic, on the other hand a historical munitions disposal site of German and British ordnance from World War II. Approximately 30,000 t of munition including torpedoes, moored mines, ground mines, aerial bombs, and depth charges were originally dumped in the area (Kampmeier et al., 2020). More than 6000 mines with over 1600 t weight represent the biggest part of dumped munition in KH (Kampmeier et al., 2020). In recent investigations, more than 1000 objects in KH were discovered using repeated high-resolution multibeam and underwater video surveys by Kampmeier et al. (2020).

Munitions contain various harmful substances which can affect marine biota like fish. One of them is mercury (Hg), which is included either as elemental Hg or Hg fulminate (a common explosive primer), and thus may act as a local source of Hg in the dumping areas (Beldowski et al., 2019). It has been shown that explosive material released from dumped munition in KH can be found in the direct vicinity of the munition in water and in different biota (Beck et al., 2018, 2019; Gledhill et al., 2019; Maser & Strehse, 2021; Strehse & Maser, 2020; Strehse et al., 2017) as well as in fish (Koske et al., 2020b). Besides explosives also compounds related to chemical warfare agents can leak from dumped munition and have already been detected in fish from the Baltic Sea (Niemikoski et al., 2020). So it is likely that besides explosives and chemical warfare agents also Hg from the munition might be released in the environment. Beldowski et al. (2019) confirmed this by detection of increased concentrations of Hg in sediments from KH. The same authors detected Hg fulminate (Hg (CNO)_2_) in sediments confirming that KH is a local point sources of Hg originating from dumped munition. Also Uścinowicz et al. (2011) observed high Hg concentrations in Baltic Sea sediments from the specific munitions dumpsites. On the other hand, Kampmeier et al. (2020) stated that mainly unfused munitions have been dumped in KH which is not likely to contain Hg-fulminate. But at least parts of the munition must have been still armed, as explosion accidents happened during the dumping work (Kampmeier et al., 2020). Therefore, markedly Hg contamination at KH caused by dumped munition can be questioned.

Hg exists as inorganic Hg and as organic Hg (primarily methylHg); it is ubiquitous in the marine environment and at the same time is considered one of the most toxic elements or substances on the planet. Hg is released from natural and anthropogenic sources (Clarkson & Magos, 2006). Direct atmospheric deposition of Hg is regarded as major source of contamination of the seas (Driscoll et al., 2013), and half of the emitted anthropogenic Hg has accumulated in the oceans and marine sediments (Zhang et al., 2015). In the environment, a variety of adverse effects in fish at physiologic, histologic, bio-chemical, enzymatic, and genetic levels can be induced by Hg (Morcillo et al., 2017), partly at environmentally realistic concentrations. Lang et al. (2017) reported higher disease prevalences in fish going alongside with enhanced Hg levels in the North Sea. Dabs (*Limanda limanda*) are suitable organisms for environmental screening due to their benthic lifestyle, geographically widespread and considered to be relatively stationary at the same time. It has been used as a bioindicator in many studies, e.g. on heavy metals (Lang et al., 2017) or organic contaminants (Kammann, 2007; Kammann et al., 2017). 

The potential environmental threat related to dumped munition has gained attention in international monitoring: The European Marine Strategy Framework Directive (MSFD) aims for establishing a good environmental status of European marine waters by 2020. MSFD names under Descriptor 8, munition disposal sites as a source for contamination and pollution (Law et al., 2010). Monitoring is the prerequisite for predictions of contamination rates, accumulation of toxic substances in biota and thus risk assessment which may lead to remediation of munition dumpsites in future.

Munition might be a relevant source of Hg for bottom dwelling fish like the dab. However, there is a knowledge gap if Hg from dumped munition enhances exposure for fish and by this might affect them—probably together with other contaminants from dumped munition. Therefore, the present study aims to answer the questions:

1. Are dab from the munition dump site KH higher contaminated with Hg than those from reference areas?
2. Can the exposure of individual fish to munition (indicated by explosives in bile) be related to Hg concentration at munition dump site?
3. Is Hg contamination of fish a suitable marker for munition exposure?

## Material and methods

### Study sites

**Fig. 1:**
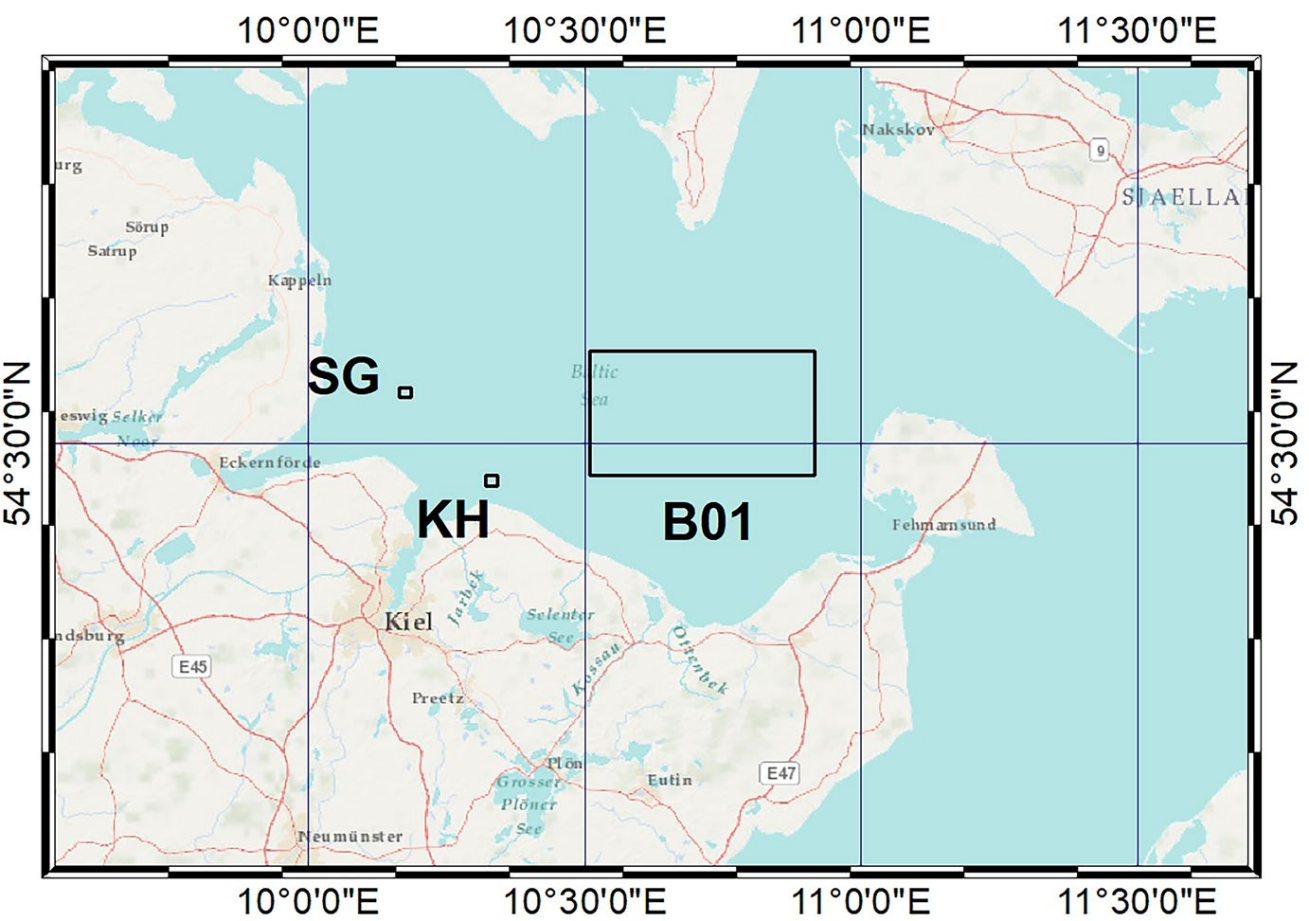
Sampling sites of dab in the western Baltic Sea: Kolberger Heide dumpsite (KH) and reference sites, B01, and Stoller Ground (SG) close to the German coastline (Sources of the basemap: Esri, Garmin, USGS, NPS

The region of all study sites is the western Baltic Sea. KH is a 1260 ha restricted munition dumpsite with approximately 30,000 t of conventional munition dumped (Gledhill et al., 2019). The reference sites were used for comparison: Stoller Ground (SG) located 10 km west of KH, B01 about 25 km northeast of KH. According to the AMUCAD database (North.io GmbH, 2019), no actual munition contamination is documented at SG. B01 is located close to the Fehmarn Belt the latter being contaminated by munition as hundreds of ground mines were dropped there during the war (Böttcher et al., 2011). The locations of the sampling sites are shown in Fig. 1; geographical coordinates are given in Table 2.

### Sampling

Dab (*Limanda limanda*) were collected during two cruises of RV Clupea (CLU314 and CLU326) in August 2017 and 2018 respectively by gillnet fishery at the edges of KH and by bottom trawling in SG (TV-300 bottom trawl, 15–20 min towing time at 3–4 knots). Additionally, dab were collected in B01 during one cruise of RV Walther Herwig III (WH408) in September 2017 by bottom trawling (140 ft. bottom trawl, 60 min towing time at 3–4 knots). Detailed cruise information is provided in Table 2. Live dab were randomly sorted from the catches and kept alive in tanks with running seawater of ambient water temperature prior to dissection. Fish were weighted, the total length measured, sex visually determined and animals anaesthetized by a blow on the head, followed by decapitation. The skin was partly removed, and a portion of muscle fillet of individual fish was collected with a ceramic knife and stored frozen in precleared plastic tubes (rinsed with nitric acid 6.5% and ultrapure water) and stored at − 20 °C until further processing. Subsequent analyses were carried out under clean lab conditions of ISO class 7.

### Condition factor and age determination

Biometric data were used to determine Fulton ‘s condition factor (CF = weight [g] * 100/length [cm]^3^) as an indicator of the general fish health status. Otoliths were removed for subsequent age determination according to Maier (1906) and Bohl (1957). All biometric data characterizing the fish under analysis are presented in Table 2.

### Chemicals

Nitric acid, 69% in ultrapure quality and certified standard solutions of Hg were purchased from Carl Roth, Karlsruhe, Germany, in 0.5 M nitric acid. Ultrapure water was obtained from a Purelab Flex 3 device (Elga Veolia; High Wycombe, UK).

### Hg measurement and quality assurance

For sample preparation, portions of muscle samples were freeze dried using a lyophilizer (LD 1–2, Christ, Osterrode, Germany) and subsequently homogenized using an agate mortar or an ultra turrax tube drive dispenser (IKA, Staufen, Germany) equipped with glass grinders respectively to obtain a dry sample powder suitable for Hg analysis. Total Hg was determined by atomic absorption spectrometry using a Direct Mercury Analyzer (DMA-80, MLS, Leutkirchen, Germany). Known amounts (20–30 mg) of each sample were weighted into the boat containers (precleaned with nitric acid) of the DMA-80. Direct analysis for total Hg content was performed using a 10-level calibration with standards in 0.5 M nitric acid. The accuracy of the procedure was determined by analysis of Certified Reference Material (DORM-3 and DORM-4, both fish protein homogenates) obtained from the National Research Council (NCR) in Canada which was taken through the same analytical procedure as the samples. Details on reference materials are given in Table 1. All samples were analysed in triplicate. External quality assurance was done by successfully participation in laboratory proficiency tests (*z*-score 0.7) conducted by QUASIMEME (www.wepal.nl) designed for marine environment analytics. The limit of detection (LD) and the limit of quantification (LQ) were calculated from a standard curve according to DIN 32,645 (DIN, 1994) with a confidence level of 99%. Considering the sample preparation a LD of 0.080 µg/kg wet weight (w. w.) and a LQ of 0.230 µg/kg w. w. were determined for Hg. No values below these limits were found in any sample under investigation. Recovery of the method was 100.3% and precision was 8.20%. All analytical results are presented in Table 2.

**Table 1:** Values of Hg in Certified Reference Materials DORM-3 and DORM-4. Measured means and standard deviations (SD) obtained accompanying sample analysis

	DORM-3, Hg	DORM-4, Hg
Assigned value [µg/kg w. w.]	382	410
Range [µg/kg w. w.]	322–442	355–465
Measured mean [µg/kg w. w.]	351	362
Measured SD	23.7	19.6
n	14	18

**Table 2:** Cruise information, biometric and contaminant data of dab caught at sites B01, Stoller Ground (SG) and Kolberger Heide (KH). Location is given as latitude and longitude for a rectangle. Total length [cm], condition factor (CF), age [y], mercury (Hg) [µg/kg] in fish muscle related to wet weight (w. w.) and explosive compound 4-aminodinitrotoluene (4-ADNT) [ng/mL] are expressed as mean values with minima and maxima in brackets. Number of individuals per sex is given. *The limit of detection (LOD) of 4-ADNT is 2.8 ng/ml. 4-ADNT results and LOD are taken from Koske et al. (2020b)

Site	Coordinates [°mm, ss}	N	Age [years]	Length [cm]	Sex f/m	CF	Hg [µg/kg w. w.]	4-ADNT [ng/ml]*
KH	54°27,28′ N–54°27,97′ N 10°19,28′ E–10°20,56′ E	99	5.1 (1–9)	29.3 (19–36)	87/12	0.98 (0.77–1.43)	66.74 (14.40–173.90)	37.5 (< LOD*-141.1)
SG	54°32,87′ N–54°33,52′ N 10°09,94′ E–10°11,23′ E	132	3.6 (2–7)	25.8 (20–35)	103/29	1.02 (0.81–2.01)	39.34 (6.64–110.56)	< LOD
B01	54°28,00′ N–54°35,80′ N 010°30,60′ E–10°55,00′ E	20	3.6 (2–6)	25.7 (21–31)	15/5	1.01 (0.80–1.20)	42.57 (17.72–92.93)	0.59 (< LOD-6.93)
All		251	4.2 (1–9)	27.2 (19–36)	204/47	1.01 (0.77–2.01)	49.80 (6.64–173.90)	14.9 (< LOD-141.1)

### Statistics

Statistical analyses were carried out using Statistica Version 12.5 (Statsoft Europe, Hamburg Germany). The correlation between concentration of Hg in muscle and the age of fish as well as between 4-ADNT in bile and Hg in muscle in fish from KH was tested using linear regression as well as by Spearman rank correlation. The principal component analysis (PCA) was performed using varimax rotation. An ANOVA (95% confidence level, 0.05 significance threshold) was conducted to investigate age and site influence on Hg concentrations.

## Results and discussion

Fish from three study sites in the western Baltic Sea (Fig. 1) were included in this study. A total of 251 dab were examined and individual muscle samples were analysed for Hg. Comparing biometric data, the dab from the munition dumpsite KH were the largest and oldest fish, while fish from the reference sites SG and B01 were smaller and younger (Table 2). At every study site, more female than male dab were caught. The mean values of the condition factor (CF) at all study sites were in the range of 0.98 to 1.02 (Table 2) with no significant differences between the sites. Therefore, CF is not likely to mirror any negative influence at a single site.

Samples from the munition dump site KH and from two reference sites in the vicinity of Kiel Bight (B01, SG) were analysed for total Hg. All samples exhibited Hg concentrations above LOQ. The maximum concentration of Hg measured in single samples was 173.90 µg/kg w. w. in a fish from KH. Highest mean concentration of Hg was also reported in KH with 66.74 µg/kg w. w. Minimum single Hg concentration of 6.64 µg/kg w. w. was determined in a sample from SG. Also lowest mean concentration of 39.34 µg/kg w. w. was calculated for SG. Table1 also contains concentrations of Hg and explosive compound 4-aminodinitrotoluene (4-ADNT) measured in the same fish (bile) but already published in Koske et al. (2020b).

**Fig. 2:**
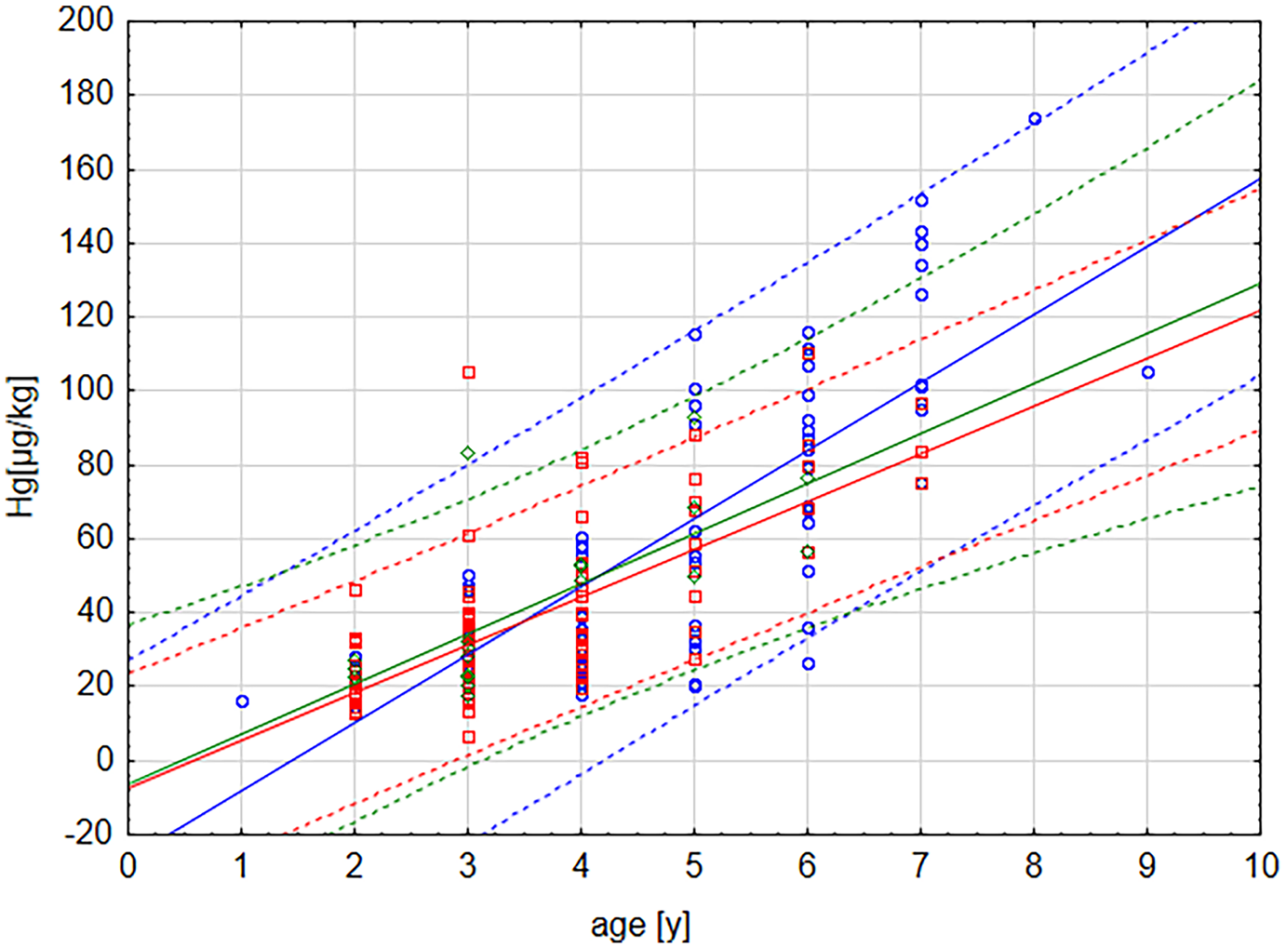
Relation between Hg concentration [µg/kg ww] in muscle and age [years] of the dabs separated by sampling site Kolberger Heide (KH, blue), Stoller Ground (SG, red) and B01 (green) in the western Baltic Sea. Given are mean correlation functions (linear, solid lines) and 95% prognosis bands (dashed lines) for each site

Figure 2 shows the relation between age of the fish and the concentrations of Hg in the three sites under investigation:

1 $$\begin{aligned}Hg[{\mu{g}/kg]}=&-26.397+18.394\\ &*{age}\;[y]\;(r=0.7628)\;for\;site\;KH,\end{aligned}$$

2 $$\begin{aligned}Hg[{\mu{g}/kg]}=&--7.415+12.942\\ &*{age}\;[y]\;(r=0.7408){\;for\;site}\;SG\;and\end{aligned}$$

3 $$\begin{aligned}Hg[{\mu{g}/kg]}=&-6.118+13.524\\ &*{age}\;[y]\;(r=0.7157)\;for\;site\;B01.\end{aligned}$$

Hg bioaccumulation in dab for all sites under investigation is described by:

4 $$Hg[{\mu{g}/kg]}=-17.503+16.217*{age}\;[y]\;(r=0.7902).$$

Correlation analysis between Hg in muscle tissue and 4-ADNT in bile of the same fish at site KH did not lead to any significant correlation, expressed by a non-significance in linear correlation (*p* > 0.05) and a low correlation coefficient *r* = 0.189. Non-linear Spearman rank-correlation leads to comparable results (results not shown).

ANOVA results on possible site and/or age effects on Hg revealed a significant correlation (*p* > 0.001) between age and Hg but no significant relation between site and Hg.

**Fig. 3:**
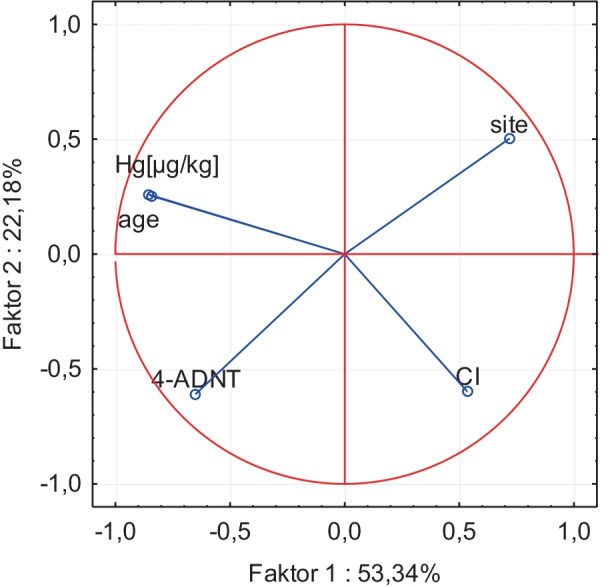
Principal component analysis of dab from three sites in the western Baltic Sea, variable projection on factors 1 and 2: *Hg* mercury in muscle tissue [µg/kg ww]; *4-ADNT* 4-aminodinitrotoluene in bile [ng/ml] according to Koske et al. (2020b); site = KH, SG or B01 (compare Fig. 1); age [years]; *CF* condition factor

The PCA in Fig. 3 explains 75.52% of the variance with the first two factors. Factor 1 explains 53.34% of the variance and refers mainly to age and Hg. Factor 2 explains 22.18% of the total variance and is dominated by 4-ADNT and CF. However, the variable site shows weaker relations to both of the first two factors. An overview on factor loadings is given in Table 3.

**Table 3:** Loadings for the first two factors (F1, F2 with variance levels) of a principal component analysis of dab from three sites in the western Baltic Sea. *Hg*, mercury in muscle tissue [µg/kg fresh weight]; *4-ADNT*, 4-aminodinitrotoluene in bile [ng/ml] according to Koske et al. (2020b); site = KH, SG or B01 (compare Fig. 1); age [years]; *CF*, condition factor. Factor loadings above/below ± 0.5 are marked in bold

	F1 (53.34%)	F2 (22.18%)
Age	** − 0.5246**	0.2465
Hg	** − 0.5168**	0.2409
Site	0.4395	0.4768
4-ADNT	** − **0.3974	** − 0.5770**
CF	0.3266	** − 0.5665**

The present study aims to analyse Hg concentrations in muscle tissue of dab from a munition dump site compared to reference sites in the western Baltic Sea. It also aims to reveal possible relationships between Hg concentrations and exposure to munition as well as to consider biological parameters like age of the fish. This contributes to the overall question if Hg in fish can act as a marker for munition exposure in future monitoring studies. The range of Hg contamination in dab muscle tissue reported in Table 2 is in well accordance with the contamination range covered by former studies (Bayens et al., 2003; Lang et al., 2017; HELCOM, 2018). Lang et al. (2015) reported Hg concentrations in muscle tissue of dab ranging from 7 to 373 µg/kg w. w. (mean 52 µg/kg w.w.).

At first sight, results displayed in Table 2 with maximum mean concentrations of Hg in samples from KH show a general higher Hg contamination in KH than in the reference sites. However, it is known that Hg in dab is mainly present as methyl mercury (94%, Lang et al., 2017), which shows bioaccumulation in fish (Donadt et al., 2021). A detailed look at the Hg concentrations and the age, shown in Fig. 2, reveals a clear correlation between Hg and age for all stations (Eq. (4)). This illustrates that age-dependent bioaccumulation of Hg in dab from all stations takes place and bioaccumulation follow comparable functions with overlapping 95%-prognosis bands in Fig. 2. Therefore, the age-Hg-relations can be regarded similar for all sites so that the typical Hg concentration of a 2-year old dab is about 19 and for a 4-year old dab is about 47 µg/kg w. w. (calculated from equation [4], shown in Fig. 2) in the southern Baltic Sea. The higher Hg concentration in fish from KH (Table 2) is therefore mainly caused by their higher age and not by higher contamination level of, e.g. sediments. This leads to the question, if earlier findings of elevated Hg concentration in KH sediments before (Beldowski et al., 2019) could explain enhanced Hg concentration of fish regarding (1) its concentration relatively to diffuse sources and (2) the biovailability of Hg-fulminate for fish. It has to be taken into account, that highest individual Hg levels were reported in fish from KH.

Koske et al. (2020b) reported for the same individuals which have been used in the present study set that only about half of the fish were tested positive for explosives (4-aminodinitrotoluol in Table 3) and therefore had a proven contact to dumped munitions. However, no indication could be found in the present study that fish determined positive for explosive compounds tend to higher Hg levels. This might be explained by the different time scales of exposure when a metabolite is detected in bile (days) compared to bioaccumulation of Hg in muscle (months/years). It is also possible that exposure to explosives and to Hg takes place in parallel in KH but that Hg contamination originating from Hg fulminate has either a low bioavailability, or a low concentration compared to other diffuse Hg sources and is therefore hard to detect.

A PCA illustrates the relations in the data set in Fig. 3 and confirms that the variables age and Hg are related as expected from Fig. 2 (displayed closely in the projection and covered by same factor, Table 3). The PCA also shows a weaker relation between variables 4-ADNT and site as described by Koske et al. (2020b) (variables inversely correlated on the same diagonal in the projection in Fig. 3). There is no clear relation between Hg and 4-ADNT because they were mainly covered by different factors (Table 2). Results of correlation analysis (Fig. 2) as well as of PCA (Fig. 3) are supported by ANOVA showing a significant correlation (*p* > 0.001) between age and Hg but not between site and Hg. All three statistical methods point in the same direction: Dumped munition at KH is not likely to be a Hg source for fish.

As Hg is a core indicator in environmental monitoring programme for the Baltic Sea, HELCOM (2018) reported in its second holistic assessment that concentrations of Hg in fish muscle exceeded the threshold level of 20 µg/kg w. w. in almost all monitored regions indicating no good status for the Baltic Sea. This is in accordance with our findings and underlines the importance of Hg measurements in the marine environment and the need of knowledge about local sources of Hg to interpret monitoring results; especially if they are related to dumped munition. The German environmental ministers decided in 2019 to initiate a process to set up a screening in munition dump sites and reference areas to assess possible environmental impact on the ecosystem originating from dumped munitions in German marine waters (UMK, 2019). Hg is discussed as one of the indicators for munition exposure besides explosives such as 4-ADNT. KH is likely to be included in this future screening study because it is a well-studied region (Kampmeier et al., 2020; Koske et al., 2020a, b; Strehse et al., 2017). For investigations described above or a later monitoring on dumped munition, e.g. under MSFD suitable indicators in fish have to be selected. As outlined before, Hg does not seem to act as suitable indicator for monitoring of dumped munition at KH.

## Conclusion

We conclude that elevated Hg levels present in dump site sediments (Beldowski et al., 2019) do not significantly influence Hg contamination in fish living there. Therefore, Hg in fish is no suitable indicator for exposure to dumped munition at KH. We hypothesize that Hg from diffuse sources may overlay the additional input at dump sites. However, Hg exposure originating from dumped munition cannot be excluded in general as local contamination source for fish and may contribute to the overall exposure monitored, e.g. under MSFD D8.

## Data Availability

The datasets generated during and/or analysed during the current study are available from the corresponding author on reasonable request.
